# Interval magnetic resonance imaging in eosinophilic fasciitis patients with discordant patient-reported symptoms and physician assessment

**DOI:** 10.1016/j.jdin.2026.08.012

**Published:** 2026-09-07

**Authors:** Abhinav Vempati, Amina Nasari, Leila Shayegan, Yoo Jung Kim, Joshua Prenner, Kathryn Rentfro, Connie Y. Chang, Ruth Ann Vleugels, Neda Shahriari

**Affiliations:** a Icahn School of Medicine at Mount Sinai, New York, New York; b Department of Dermatology, Harvard Medical School, Brigham and Women's Hospital, Boston, Massachusetts; c City University of New York School of Medicine, New York, New York; d Division of Dermatology, Lahey Hospital and Medical Center, Burlington, Massachusetts; e Department of Dermatology, Mayo Clinic, Rochester, Minnesota; f Department of Radiology, Harvard Medical School, Massachusetts General Hospital, Boston, Massachusetts

**Keywords:** eosinophilic fasciitis, fascial enhancement, fascial thickening, magnetic resonance imaging, steroid escalation

*To the Editor:* Eosinophilic fasciitis (EF) is a rare sclerosing disorder clinically characterized by painful skin induration, limb swelling, and peripheral eosinophilia involving underlying fascial inflammation.^1^ The gold standard for the diagnosis of EF is an incisional wedge biopsy. However, more recent studies have demonstrated the non-inferiority of magnetic resonance imaging (MRI) in EF detection.^2^ In particular, characteristic MRI findings of active EF include fascial thickening with hyperintensity on T2-weighted imaging and post-contrast T1-weighted enhancement.^3^ Treatment of EF includes high-dose systemic steroids, with a slow taper, alongside a steroid-sparing agent, though dose escalation of steroids is often recommended for acute flares and relapses necessitating proper interval disease assessment.^4^ Currently, evaluation of EF disease progress is dependent on physician clinical assessment. Discordance between subjective patient symptoms and physician clinical assessment poses a management and therapeutic dilemma in EF.

There is limited data evaluating the utility of MRI in assessing EF progress. One study evaluated MRI in demonstrating resolution of fascial inflammation in 6 patients treated with EF, corresponding to clinical remission.^5^ We performed a retrospective cohort study of patients with EF treated at Mass General Brigham (MGB) between January 1, 2000 and June 1, 2025. Patients were identified via International Classification of Disease (ICD-10) codes and natural-language query and were included if they received both baseline and repeat MRI at follow-up. This study was approved by the MGB institutional review board.

We identified 127 patients with EF, of whom 52 had undergone a baseline MRI. Nineteen patients had repeat MRI and were included in this study. Their demographics are presented in Table I. MRI was performed when subjective symptoms of flare—pain, tightness, or swelling (16/19, 84.2%)—or subjective lack of clinical improvement (3/19, 15.8%) was noted by patients but the physician’s clinical assessment did not favor worsening EF. In 17 patients (89.5%), MRI demonstrated either no/minimal active fasciitis or reduced fascial inflammation relative to baseline, corroborating physician clinical assessment. In these patients, clinicians continued steroid tapers or maintained stable treatment regimens. Detailed MRI findings and treatment management are available in Table II. Patients were monitored long-term (at least 12 months) and 16 (94.1%) remained clinically stable without documented EF flare. In 2 patients in the study, active inflammation was noted on MRI leading to steroid dose escalation.

**Table I:** Characteristics of study population

	No. (%)
No. of patients	19 (100)
Sex	
Female	12 (63.2)
Male	7 (36.8)
Age, median, y	68
Race	
White	17 (89.4)
Black	1 (5.3)
Asian	1 (5.3)
Time to MRI	
Median time to MRI (range), mo	17.3
Maximum time to MRI, mo	107.3
Minimum time to MRI, mo	4
MRI Locations	
Right upper extremity	5 (26.3)
Left upper extremity	3 (15.8)
Right lower extremity	2 (10.5)
Left lower extremity	4 (21.1)
Both lower extremities	3 (15.8)
Right upper and right lower extremities	2 (10.5)

*EF*, Eosinophilic fasciitis; *mo*, months; *MRI*, magnetic resonance imaging; *y*, years.

**Table II:** Patient outcomes following MRI

	Patient subjective symptoms	
	Possible Flare (pain, tightness, swelling)	Lack of Clinical Progress
N (%)∗	19 (100)	
Steroid dose escalation	2 (10.5)	
Active inflammation on MRI	2 (10.5)	-
No additional steroid use	17 (89.5)	
No to minimal active inflammation on MRI, continued steroid taper and/or maintained current dose	5 (26.3)	-
No to minimal active inflammation on MRI, maintained current steroid-sparing† agent dose	6 (31.6)	1 (5.3)
No active inflammation, patient remained off therapy	1 (5.3)	-
Inflammation present but improved relative to baseline, continued steroid taper and/or maintained current dose	2 (10.5)	-
Inflammation present but improved relative to baseline, maintained current steroid-sparing† agent	-	2 (10.5)‡

∗ All patients were followed for at least 12 months following interval MRI assessment.

† Steroid sparing agents included methotrexate, mycophenolate mofetil, upadacitinib, tofacitinib, IVIG, mepolizumab.

‡ One patient from this group (5.9%), maintained on tofacitinib, developed flare 6 months post MRI evaluation.

We present a cohort of patients who underwent interval MRI assessment following discordance between patient subjective symptoms of disease and physician clinical impression. This is the largest cohort to date of EF patients undergoing MRI interval assessment. For the majority of patients, MRI findings were consistent with pre-imaging physician impression, which suggests potential future clinical utility. Further studies are needed to understand the role of interval MRI in EF disease assessment. Limitations of this study include its retrospective nature and potential for selection bias.

### Declaration of generative AI and AI-assisted technologies in the writing process

No AI or AI-assisted technology was used for this manuscript.

## Conflicts of interest

None disclosed.
